# Multi-Objective White Shark Optimizer for Global Optimization and Rural Sports-Facilities Location Problem

**DOI:** 10.3390/biomimetics10080537

**Published:** 2025-08-15

**Authors:** Yan Zheng, Bin Guo, Yongquan Zhou

**Affiliations:** 1 Department of Science and Technology Teaching, China University of Political Science and Law, Beijing 100088, China; zhouguo@cupl.edu.cn; 2 College of Artificial Intelligence, Guangxi Minzu University, Nanning 530006, China; yongquanzhou@126.com; 3 Guangxi Key Laboratories of Hybrid Computation and IC Design Analysis, Nanning 530006, China

**Keywords:** multi-objective white shark optimizer, benchmark functions, global optimization, rural sports-facilities location, intelligence optimization

## Abstract

A swarm intelligence optimization algorithm called white shark optimizer (WSO) has been proposed and successfully applied in regard to many aspects. In this paper, the location problem of sports facilities is regarded as a multi-objective problem, and the number of residents covered by sports facilities and the Weber problem are introduced as objective functions. A multi-objective white shark optimizer (MOWSO) is proposed, and MOWSO introduced an archived mechanism to store the non-dominated solutions obtained by the algorithm. When the Pareto solutions in the archive overflow, the solutions are removed by calculating the true distance of the Pareto optimal solution. The performance of the MOWSO is verified on CEC 2020 benchmark functions, and the results show that the proposed MOWSO is better than other algorithms in the diversity and distribution of solutions. The MOWSO is applied to solve the rural sports facilities location problem, and a variety of different sports facilities location schemes are obtained. It can provide a variety of options for the location of rural sports facilities, and promote the intelligent design of sports facilities.

## 1. Introduction

The World Health Organization (WHO) advocates that humans can promote physical health and develop a healthy lifestyle through appropriate physical exercise, but progress is slow at present [1]. With rapid urbanization, China faces problems of obesity and a lack of physical exercise. Therefore, how sports are promoted in China is gaining people’s attention more and more [2]. One of the core aspects of China’s sports policy is to build a viable environment to promote people’s participation in sports activities in a way that is consistent with the health-promotion strategy advocated by the World Health Organization [3]. Compared with developed countries, the average level of sports facilities in China is relatively backward. Compared with the city, the average level of rural sports facilities has an obvious lag, and the number is small, unable to meet the needs of the majority of residents for physical exercise, which has become one of the factors restricting the physical fitness training of villagers. The above situation can be improved by promoting the process of rural sports development, and the construction of rural sports facilities is the key to promoting the development of rural sports. The state promotes the implementation of the rural revitalization strategy by increasing the capital investment in the construction of rural sports facilities. In the process of the construction of rural sports facilities, how to choose the right location for the construction of rural sports facilities is one of the key problems in the construction of rural sports facilities. The construction of sports facilities can promote the physical exercise of rural residents, improve the physical fitness of residents, contribute to the sustainable development of rural areas, and promote the implementation of rural revitalization strategies. Based on the classical continuous location model, this paper constructs a multi-objective location model of rural sports facilities from the perspectives of fairness and efficiency.

Over the past few decades, different meta-heuristic algorithms have been developed to handle these complex optimization problems, such as differential evolution (DE) [4,5,6,7], particle swarm optimization (PSO) [8,9,10,11], flower pollination algorithm (FA) [12], ant colony optimization (ACO) [13,14], sine cosine algorithm (SCA) [15,16], gray wolf optimization (GWO) [17,18,19], Tianji’s horse racing optimization (THRO) [20], the Divine Religions Algorithm (DRA) [21], and Stellar oscillation optimizer (SOO) [22], etc. It is proven that the metaheuristic algorithm has important application value. In academia and industry, these algorithms are widely used to solve various types of optimization problems. Because of their simple principle and easy implementation, these algorithms have attracted more and more attention. Unlike traditional methods to solve optimization problems, metaheuristic algorithms do not need to analyze objective functions and constraints, which is the most significant advantage of metaheuristic algorithms. In general, a metaheuristic algorithm starts with a random set and then searches globally for the best solution according to the set rules. These rules often mimic the laws of natural evolution, the laws of human behavior [23], the social behavior of birds, insects, and other organisms [24,25], the laws of plant growth [26], mathematical planning [27], and the laws of physics [28,29], etc. The literature review is summarized in Table 1.

Multi-objective optimization is a common problem in all fields. In multi-objective problems, each objective will not reach the optimal solution at the same time. It is important to find multiple sets of solutions for multi-objective problems because different solutions can be applied to decision-makers with different needs. In recent years, many meta-heuristic algorithms have been successfully applied to multi-objective optimization problems. One of the very classic and popular multi-objective algorithms is multi-objective particle swarm optimization (MOPSO) [30]. In MOPSO, the optimal Pareto solution is selected by external archiving and the adaptive grid mechanism. K. Deb improved NSGA by introducing fast elite non-dominated sorting to obtain NSGA-II [31,32]. The algorithm proposes a fast non-dominated ranking to improve the population level. In addition, crowding ranking and elite selection strategies are introduced. There are other classical multi-objective algorithms, such as SPEA2 [33] and Omni-optimizer [34], etc. In addition, there are some excellent multi-objective algorithms, such as DN-NSGAII, MO_Ring_ MO_Ring_PSO_SCD, MOHHO, MOFOA, MaODA, DPDCA, and so on [35,36,37,38,39]. These multi-objective algorithms can solve multi-objective optimization problems well. A new swarm intelligence optimization algorithm called white shark optimizer (WSO) was proposed by Malik B. et al. in 2022 [40]. Inspired by the great white shark’s search for food and tracking of prey in the deep sea, it combines the great white shark’s keen hearing and smell abilities with fish behavior. Based on the mathematical modeling of great-white-shark hunting behavior, the great-white-shark population can search through random search, locate the behavior of prey and fish, and search in the search space, so as to update position and finally achieve the purpose of searching for the best. At present, the white shark optimizer algorithm has been applied to some optimization problems and has achieved good results [41,42,43,44,45,46]. In this paper, the white shark optimizer is improved to the multi-objective white shark optimizer (MOWSO) to solve the multi-objective problem.

The following is a summary of the principal achievements:

(1) A novel multi-objective white shark optimizer (MOWSO) is proposed;
(2) The archived mechanism is introduced in MOWSO to store the current Pareto optimal solution, and it is screened by calculating the true distance of the Pareto optimal solution;
(3) The performance is tested and compared to MOWSO on CEC2020 benchmark functions;
(4) A novel rural sports-facilities location problem model is proposed;
(5) We used MOWSO to solve the rural sports-facilities location problem and obtained the best result.

The rest of this article is divided into four parts. Section 2 is the introduction of the white shark optimizer algorithm and location problem. In Section 3, the multi-objective problem is introduced, and the multi-objective white shark optimizer algorithm is designed. Section 4 evaluates the performance of the multi-objective white shark optimizer algorithm on the test set. Section 5 introduces the rural sports-facility location model and uses the white shark algorithm to solve it. Section 6 is the summary of this paper and future work.

## 2. Related Work

### 2.1. White Shark Optimizer

The white shark is one of the most ferocious and dangerous predators in the ocean. It has powerful muscles, keen eyesight, and a keen sense of smell. When searching for prey, great white sharks first use their hearing to scout large spaces. Second, they can find prey by using their keen sense of smell. These features help them explore nearby areas. Great white sharks can use two lines on their sides to detect changes in water pressure and thus find the direction of prey movement. Great white sharks sense changes in pressure in the ocean and move toward prey. They can also sense the weak electromagnetic field generated by the movement of prey. When locating prey, the great white shark moves toward it in a fluctuating motion. The algorithm is divided into three parts: speed of movement towards prey, movement towards optimal prey, and clustering behavior of fish.

#### 2.1.1. Movement Speed Towards Prey

White sharks spend most of their time hunting and tracking prey. They track their prey in a variety of ways, using their superior sensory abilities like hearing, sight, and smell. When white sharks sense the location of their prey based on the hesitation of the waves they hear as the prey moves, they move toward the prey in an undulating manner. This mode of motion can be defined as shown in Equation (1).

(1) $$v_{k+1}^{i}=\mu [v_{k}^{i}+p_{1}(w_{gbest_{k}}-w_{k}^{i})\times c_{1}+p_{2}(w_{best}^{v_{k}^{i}}-w_{k}^{i})\times c_{2}]$$

where $v_{k+1}^{i}$ represents the speed of the i-th white shark to step k+1 in the white-shark population. μ represent the contraction factor, which is used to analyze the convergence behavior of great white sharks, and its definition is shown in Equation (5). $v^{i}$ is the i-th indicator vector when the white shark searches for the best prey, and its definition is shown in Equation (2). $p_{1}$ and $p_{2}$ are used to control the influence coefficients of $w_{gbest_{k}}$ and $w_{best}^{v_{k}^{i}}$ on $w_{k}^{i}$, and their calculation methods are shown in Equations (3) and (4), respectively. $w_{gbest_{k}}$ is the global optimal position obtained by the white shark in the first *k* searches so far in the search process, $w_{best}^{v_{k}^{i}}$ is the optimal position corresponding to the i-th white shark in the population in the *k* iterations, and $w_{k}^{i}$ is the position of the i-th white shark in the population at the *k* searches. $c_{1}$ and $c_{2}$ are randomly generated numbers between 0 and 1. i represents the i-th white shark in the white-shark population.

(2) $$v=\left\lfloor n\times rand(1,n)+1\right\rfloor$$

where $rand\left(1,n\right)$ is a uniformly distributed random number generated in the range of 0 to 1.

(3) $$p_{1}=p_{\max}+(p_{\max}-p_{\min})\times e^{-{\left(4k/K\right)}^{2}}$$

(4) $$p_{2}=p_{\min}+(p_{\max}-p_{\min})\times e^{-{\left(4k/K\right)}^{2}}$$

where *k* and *K*, respectively, represent the number of iterations and the maximum number of iterations of the white-shark population so far. $p_{\min}$ and $p_{\max}$ are two fixed values set empirically, 0.5 and 1.5, respectively, in order to control the $p_{1}$ and $p_{2}$ values in combination with the number of iterations.

(5) $$\mu =\frac{2}{\left|\left.2-\tau -\sqrt{\tau^{2}-4\tau }\right|\right.}$$

where τ represents the coefficient of acceleration, thorough analysis results in a value of 4.125.

#### 2.1.2. Movement Towards Optimal Prey

White sharks are constantly shifting their position, and when they hear waves from prey movement or smell prey, they move closer to their prey. The location of the prey changes as it searches for food or the white shark moves towards it. However, the prey will leave some scent in the previous location. In this case, white sharks can search for prey based on the scent they left behind. This behavior of the white shark can be described in Equation (6).

(6) $$w_{k+1}^{i}=\left\{\begin{array}{l} w_{k}^{i}\cdot \neg ⊕w_{o}+u\cdot a+l\cdot b;\;\;\;\;\;rand<mv\\ w_{k}^{i}+v_{k}^{i}/f;\;\;\;\;\;rand\geq mv \end{array}\right.$$

where $w_{k+1}^{i}$ represent the latest position of the i-th white shark in the population after the (*k* + 1) iteration, ¬ indicates a logical non-operation, a and b are binary vectors whose formulas are Equations (7) and (8), and u and l represent the upper and lower bounds of the entire search space, respectively. $w_{o}$ represents a logical vector calculated by Equation (9), and f represents the frequency of the wave motion of the white shark, and the formula is represented by Equation (10). rand represents a random number between 0 and 1, and mv represents the motion force of a white shark approaching prey, which increases with the number of iterations and which is calculated by Equation (11).

(7) $$a=\mathrm{sgn}(w_{k}^{i}-u)>0$$

(8) $$b=\mathrm{sgn}(w_{k}^{i}-l)<0$$

(9) $$w_{o}=⊕(a,b)$$

where ⊕ stands for the XOR operation.

(10) $$f=f_{\min}+\frac{f_{\max}-f_{\min}}{f_{\max}+f_{\min}}$$

where $f_{\max}$ and $f_{\min}$ represent the maximum and minimum frequency of the great white shark’s fluctuating movement, respectively. By conducting a large number of experimental tests, $f_{\max}$ and $f_{\min}$ were finally selected as 0.75 and 0.07, respectively.

(11) $$mv=\frac{1}{\left(a_{0}+e^{(K/2-k)/a_{1}}\right)}$$

where $a_{0}$ and $a_{1}$ are constants used to manage exploration and exploitation capabilities. Typically, the values are 6.25 and 100.

#### 2.1.3. The Swarming Behavior of White Sharks

When great white sharks hunt, they cooperate and move toward the white shark closest to their prey. This behavior is represented by Equation (12).

(12) $${w^{\prime }}_{k+1}^{i}=w_{gbest_{k}}+r_{1}{\overset{⃑}{D}}_{w}\mathrm{sgn}(r_{2}-0.5)\;\;\;\;r_{3}<s_{s}$$

The ${w^{\prime }}_{k+1}^{i}$ is the i-th update of the position of the white shark relative to prey. The $\mathrm{sgn}(r_{2}-0.5)$ value is 1 or −1, indicating the search direction. $r_{1}$, $r_{2}$, and $r_{3}$ represent random numbers between 0 and 1. ${\vec{D}}_{w}$ is the distance between the white shark and its prey, calculated by Equation (13). $s_{s}$ represents the olfactory- and visual-intensity parameters of a great white shark following other great whites approaching prey, as calculated by Equation (14).

(13) $${\overset{⃑}{D}}_{w}=\left|rand\times (w_{gbest_{k}}-w_{k}^{i})\right|$$

where rand is a random number between 0 and 1.

(14) $$s_{s}=\left|1-e^{\left(-a_{2}\times k/K\right)}\right|$$

where $a_{2}$ is a constant, and through extensive experimental analysis, the value has been taken as 0.0005.

White sharks are highly social animals that prefer to hunt in groups. The fish behavior of white sharks is represented by Equation (15)

(15) $$w_{k+1}^{i}=\frac{w_{k}^{i}+{w^{\prime }}_{k+1}^{i}}{2\times rand}$$

where rand is a random number between 0 and 1. White sharks can update their location based on the nearest white shark to their prey.

### 2.2. Continuous Facility Location

There are many kinds of location problems, which exist in different disciplines, and different disciplines have different descriptions of location problems. However, according to the description of a location problem from different disciplines, the most essential definition of a location problem is given a metric space and the location of demand points in the space, and the location of new facilities in the space, a certain goal between facility points and demand points (generally called customers) can be optimized. This problem has an important position in real life. For more than half a century, with the development of mathematical tools, such as the introduction of optimization techniques to the problem of facility location, the field has entered a period of flourishing. People have conducted in-depth research into and analysis of facility-location problems in different fields, established optimization models and studied theoretical properties, proposed effective numerical algorithms to solve the models, and used the research results to solve practical problems.

The study of location problems spans numerous research fields such as management, engineering, geography, economics, computer science, and mathematics, etc. Its typical application is the location of factories, warehouses, hospitals, retail stores, and in addition, it is also used in fire centers, fire stations, gas stations, exploration oil wells, missile warehouses, and so on. From the perspective of decision makers, site selection is of great significance. Facility location has been accompanied by the development of human history; it has an important and profound impact on human life and production activities.

The rural location problem can be classified in different ways. According to the topology of the metric space, the location problem can be divided into discrete location, continuous location, and network location. Discrete location is the location of a given series of discrete points in the metric space. Continuous siting has a long history. New facilities in continuous siting can be located in a continuous region of the metric space. Continuous optimization, such as linear and nonlinear programming, can be used to solve the continuous siting problem. Network location is the location of the vertex or edge of a given graph or network, which is solved by combinatorial optimization and integer programming. Discrete location, continuous location, and network location have important applications in real life, which can be used to solve practical problems in different situations. The following are several key elements in continuous facility siting: the number of new facilities, the distance metric function, and the objective function, etc.

#### 2.2.1. Number of Facilities

In the facility-location problem, the number of new facilities can be one facility point or multiple facility points. According to the different number of facility points, the problem of facility location can be divided into single-facility location and multi-facility location. In general, the need to locate multiple facilities is more difficult than the need to locate only one facility. This is because in the multi-facility location problem, it is necessary to consider to which facility point the customer goes to obtain the service, or the fact that, in some cases, a particular amount of service is required by the customer, but the amount of service provided by the facility cannot meet the conditions.

#### 2.2.2. Distance Metric Function

There are various distance measurement functions in the field of location selection. One of the important distance measurement functions is the norm. It satisfies positive definiteness, homogeneity, and subadditivity, and its measured distance reflects the actual distance more truly. The LP-norm is a distance metric commonly used in continuous siting. When *p* = 1, this norm measures the L1-distance, which is often used to represent the distance between two points in a city. When *p* = 2, this norm measures the Euclidean distance, which represents the straight-line distance between two points. The distance measured by this norm when *p* = ∞ is often referred to as the Chebyshev distance. Some generalized LP-norms are also commonly used to measure the distance between a facility and a customer. When customers in the location of continuous facilities occupy a large range in the metric space, they should not be simply regarded as points but as regions. The distance between the facility and the customer becomes the distance between the point and the area.

#### 2.2.3. Correlation Function of Location Problem

According to different objectives, the functions of facility location can be roughly divided into pull objectives, push objectives, and push-and-pull objectives [42,43]. When the facility is of the type desired by the demand point (such as a supermarket, bank, fire station, etc.), the demand point always wants the facility as close to it as possible. Therefore, pull goals are often used, such as minimizing the distance between the demand point and the facility and maximizing market share. When the facility belongs to the type that is excluded by the demand point (such as polluting factories, garbage disposal stations, etc.), the demand point wants the facility to be as far away from itself as possible. Push goals are often used, such as maximizing the sum of distances between customers and facility points, minimizing the number of customers covered by the facility, and so on. In some cases, the type of facilities is between expectation and exclusion, and there will be two contradictory or opposite factors in the problem of attraction and exclusion. This kind of location problem often adopts push–pull goals.

The minisum function is a commonly used pull target to minimize the distance between the facility and the customer. The corresponding location problem is called the minisum problem. The Weber problem is the minisum problem, where the number of facilities is one. The problem with more than one facility is called the multi-facility minisum problem, which includes two kinds of important problems: the multi-facility Weber problem and the multi-source Weber problem. The minimax function is another commonly used pull goal that minimizes the maximum distance between the client and the setup, and the corresponding model is called the minimax problem. Because the model focuses on the farthest customers, it reflects fairness to some extent. The coverage problem is also an important problem in the use of pull targets, and the coverage problem includes two types of problems: maximum coverage and minimum coverage. The former is to make facilities cover as many customers as possible when the number of facilities is given, while the latter is to cover all customers with the least facilities when the number of facilities is variable. Problems such as locating a point on the network to maximize its weighted distance to the node are called maxisum problems [47]. Like pull targets, the maxisum and maximin functions are two important types of push targets. The maxisum problem seeks to maximize the distance between the customer and the facility, while maximin seeks to maximize the closest distance between the customer and the facility. When the number of facilities is more than one, the multi-facility model of the push target can be divided into max–min–min, max–min–sum, max–sum–min, and max–sum–sum, etc., in which min/sum of the third layer represents the minimum distance or distance sum between a given customer and all facilities. Because of the attraction and repulsion of push–pull targets, the location problem of push–pull targets can be modeled as two-objective optimization. Therefore, the method and technology of double objective optimization can be effectively used to solve the location problem of push–pull targets.

## 3. Multi-Objective White Shark Optimizer

### 3.1. Multi-Objective Optimization Problems

Multi-objective optimization is a common problem in real life, which refers to the realization of multiple objectives under certain circumstances. However, in a multi-objective problem, each object contradicts each other, and when one object tends to the optimal value, the value of the other object may be weakened. That is, all the objects in the multi-objective problem cannot obtain the optimal solution at the same time. Solving this problem usually involves finding a balance between multiple goals, making compromises, and making each goal as relatively optimal as possible. The multi-objective optimization problem can be expressed by the following formula [48].

(16) $$\begin{array}{cc} Minimize: & F(x)=\{f_{1}(x),f_{2}(x),\ldots ,f_{o}(x)\}\\ Subject\;to: & g_{i}(x)\geq 0,\;\;\;\;\;\;\;\;\;\;\;\;i=1,2,\ldots ,m\\ & h_{i}(x)=0,\;\;\;\;\;\;\;\;\;\;\;\;i=1,2,\ldots ,p\\ & L_{i}(x)\leq x_{i}\leq U_{i},\;i=1,2,\ldots ,n \end{array}$$

where o represents the number of objective functions, m and p represent the number of inequality constraints and equality constraints, respectively, and n represents the number of independent variables. $\left[L_{i},U_{i}\right]$ are the upper and lower bounds of the i-th variable.

The concept of Pareto optimality is widely used in the search of multi-objective optimization algorithms. Pareto optimality is an ideal situation for resource allocation. Pareto improvement is a change from one allocation state to another, assuming that there are fixed units and resources that can be allocated, without reducing the resources allocated to any unit, so that the resources allocated to another unit are increased. Pareto optimality means that there are no more Pareto improvements. Here are some concepts and terms about Pareto optimality.

**Pareto domination:** If $x_{A}$ and $x_{B}$ are two solutions to a multi-objective optimization problem, $x_{A}$ performs no worse than $x_{B}$ on any objective function, and $x_{A}$ performs better than $x_{B}$ on some objective function, $x_{A}$ is said to be Pareto superior to $x_{B}$.

(17) $$\forall i\in \{1,2,\ldots ,o\},f_{i}(x_{a})\leq f_{i}(x_{b})\wedge \exists i\in \{1,2,\ldots ,o\},f_{i}(x_{a})<f_{i}(x_{b})$$

**Pareto optimal solution**: When there is no solution that dominates x, the solution to x is a Pareto optimal solution.

(18) ∃xo¬∈Xf:fi(xo)<fi(x)

**Pareto optimal solution set:** The set of all Pareto solutions is called the Pareto solution set, to wit,

(19) $$PS=\{x{|}^{\neg }\exists x_{o}\in X_{f}:f_{i}(x_{o})<f_{i}(x)\}$$

**Pareto optimal frontier:** The Pareto frontier is formed when all the Pareto optimal solutions are mapped by the objective function.

(20) $$PF=\{F(x)=(f_{1}(x),f_{2}(x),\ldots ,f_{o}(x))|x\in PS\}$$

### 3.2. The Proposed Method

The white shark optimizer algorithm can be improved by introducing two components into the multi-objective white shark optimizer algorithm. The first component is the Pareto archive, which holds the best Pareto solutions found so far [30]. The archive has rules for storing and deleting Pareto solutions, and compares the newly generated Pareto solutions with those existing in the archive to determine whether the newly generated solutions are better than the ones in the archive. When the new solution is superior to the existing solution in the archive, the new solution is not dominated by other solutions, and the new solution is stored in the archive; otherwise the new solution is discarded.

**Figure 1:**
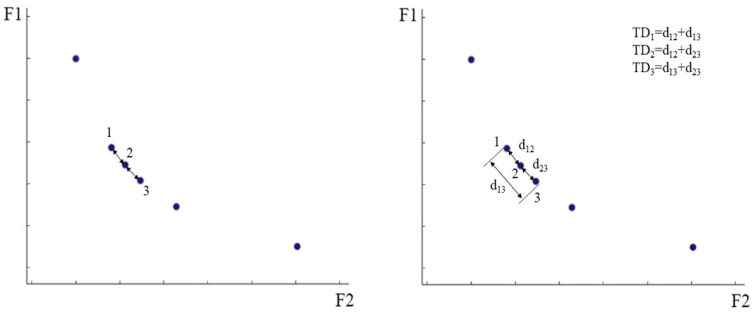
The true distance of the solution.

**Figure 2:**
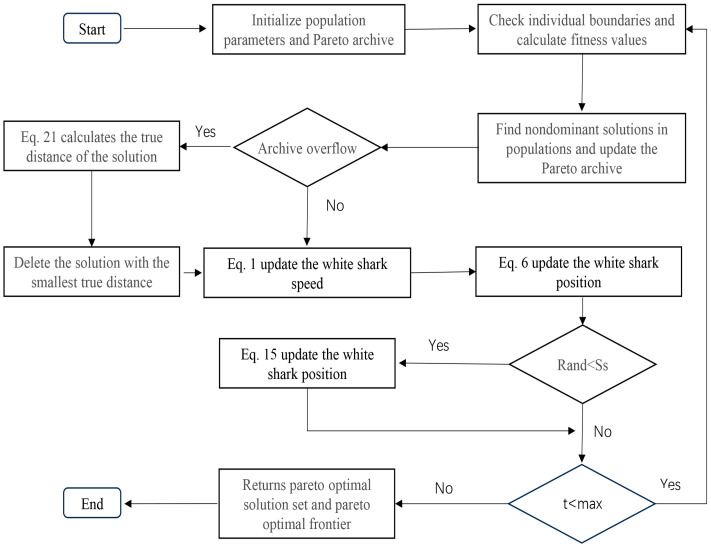
Flow chart of the MOWSO.

When the number of solutions in the archive reaches the maximum capacity of the archive, some solutions in the archive need to be deleted to make the solution set distributed more evenly. The second component is introduced at this point. The component deletes the solution according to the true distance between the solution sets. Since the solutions in the archive are all Pareto optimal solutions, it is necessary to delete some densely distributed solutions to make the distribution of solutions more uniform. In this case, the distance of each solution to its nearest solution is calculated, and then the solution with the smaller distance is preferentially deleted, but there will be cases where the minimum distance of several solutions is equal. At this point we calculate the distance between these solutions and the other closest solutions, and add these distances until we can identify the solution that needs to be specifically deleted, which we call the true distance (TD). As shown in Figure 1, when the minimum distance of solution 1, solution 2 and solution 3 is almost equal, we can calculate the true distance of each solution and delete the solution more conveniently. The calculation formulas are shown in Equations (21) and (22). As the algorithm runs, solutions with smaller true distances are removed in turn according to the second component. In addition, MOWSO guides other individuals towards the individual by selecting the one with the greatest true distance in the current archive. This method can lead the search for the optimal Pareto solution of the population, and can ensure the convergence and diversity of understanding. The flow chart of MOWSO is shown in Figure 2.

(21) $$D(i)=\sqrt{\sum_{F=1,}^{n}{\left(x_{Fi}-x_{Fj}\right)}^{2}}\;\;\;\;\;\;\;i,j=1,2,\ldots N;i\neq j$$

(22) $$TD=\sum_{p=1}^{q}D(p)$$

where n represents the number of objective functions and *N* represents the number of Pareto solutions. $x_{Fi}$ represents the value of one set of solutions, and $x_{Fj}$ represents the value of the j-th set of solutions. p represents the index of the smaller value in D.

## 4. Experimental Results and Analysis

### 4.1. Experimental Setup

All algorithms in this experiment were programmed in MATLAB R2021b and numerical experiments were performed on an AMD Ryzen 7 6800H processor with 32 GB of memory. The proposed MOWSO algorithm is compared with other classical multi-objective algorithms to evaluate its effectiveness. It includes popular multi-objective optimization algorithms NSGAII [31] and SPEA2 [33], the multi-objective particle swarm algorithm (MOPSO) [30], the generic evolutionary algorithm (Omni-optimizer) [34], and the decision space base niching NSGAII (DN-NSGAII) [32]. MOPSO was proposed by Coello C. et al. The algorithm mainly uses the archive controller and adaptive grid mechanism. DN-NSGAII is modified from NSGAII. It embeds niche techniques in mating and environmental selection schemes. Omni-optimizer is designed to solve different types of optimization problems including single-objective, multi-objective, single-modal and multimodal problems. Unlike these multi-objective algorithms, MOWSO selects the Pareto solution set based on the true distance of the solution set. When archive files overflow, the solution with the smallest true distance in the solution set is deleted. These strategies can improve the performance of the MOWSO algorithm, and can solve multi-objective problems well.

### 4.2. Evaluation Index

In this paper, the performance of MOWSO and other comparison algorithms is evaluated by using four different evaluation indexes from decision space and target space. In the decision space, Pareto Sets Proximity (PSP) [49] and Inverted Generational Distance (IGD) [50] are used, respectively. In objective space, Hypervolume (HV) is used [51], and IGD is used in objective space (IGDF) [52]. The larger the PSP and HV, the better the algorithm performance; the smaller the IGDX and IGDF, the better the algorithm performance. In order to unify, 1/PSP and 1/HV are used to evaluate the performance of the algorithm. The smaller the value of 1/PSP and 1/HV, the better the performance of the algorithm.

Pareto Sets Proximity (PSP) reflects the similarity between the true Pareto solution set (PSs) and the PSs obtained by the algorithm.

(23) $$PSP=\frac{CR}{IGDX}$$

where CR represents the coverage rate of the optimal solution set obtained by the solution to the reference solution set. The calculation formula is as follows.

(24) $$CR={\left(\prod_{l=1}^{n}\delta_{l}\right)}^{1/2n}$$

(25) $$\delta_{l}=\left\{\begin{array}{ll} 1 & V_{l}^{\max}=V_{l}^{\min}\\ 0 & v_{l}^{\min}\geq V_{l}^{\max}||v_{l}^{\max}\leq V_{l}^{\min}\\ {\left(\frac{\min(v_{l}^{\max},V_{l}^{\max})-\max(v_{l}^{\min},V_{l}^{\min})}{V_{l}^{\max}-V_{l}^{\min}}\right)}^{2} & otherwise \end{array}\right.$$

where n is the dimension of the decision space, and where $v_{l}^{\max}$ and $v_{l}^{\min}$ are the maximum and minimum values of the PSs obtained by the algorithm on the $l^{th}$ variable. $V_{l}^{\max}$ and $V_{l}^{\min}$ are, respectively, the maximum and minimum values of the true PSs obtained by the algorithm in the $l^{th}$ variable.

The index IGDX is used to judge the diversity and convergence distribution of the solution obtained by the algorithm [53]. IGDX is calculated according to the Euclidean distance between the solution obtained by the algorithm and the reference solution, which is publicized as follows:

(26) $$IGDX(O,P^{*})=\frac{\sum_{v\in P^{*}}d(v,O)}{\left|P^{*}\right|}$$

where $P^{*}$ is the true PSs and O represents the solution obtained by the algorithm. $d\left(v,O\right)$ represents the solution to the minimum Euclidean distance between the set v and O. The IGD index can also be found using the Pareto front (PF), so that the convergence distribution of the solution in the target space can be obtained. The calculation formula of IGDF is the same as that of IGDX, except that the space is different.

(27) $$IGDF(O,P^{*})=\frac{\sum_{v\in P^{*}}d(v,O)}{\left|P^{*}\right|}$$

In the objective space, the reciprocal of the hyper volume is also an important index, and its calculation formula is as follows:

(28) $$HV(S)=\underset{v\in S}{\cup }vol(v)$$

HV can measure and evaluate the convergence and diversity of the resulting solutions.

### 4.3. Experimental Result

In this paper, the performance of MOWSO was tested used CEC 2020 multi-modal multi-objective optimization benchmark functions. CEC 2020 contains 24 test functions, including different types of Pareto optimal frontiers, such as convex, concave, linear, non-linear, and disconnected. For each function, all algorithms were run independently 20 times, and the results were counted, choosing the mean and standard deviation to compare the algorithms. Table 1, Table 2, Table 3 and Table 4 shows the comparison between MOWSO and other multi-objective algorithms for the mean and standard deviation of the four indicators. Table 2 shows the 1/PSP index of different algorithms, from which it can be seen that MOWSO can find the optimal value in 14 functions, whether it is mean or standard deviation, and outperforms other algorithms. And in the other five functions, the optimal value of the mean can be found. This suggests that the Photoshop obtained by MOWSO is more like real Photoshop. For IGDX indicators, it can be seen from Table 3 that MOWSO can find the optimal value of the mean value in all 19 functions, and the performance is better than other algorithms. For the IGDF index, it can be seen from Table 4 that MOWSO can find the optimal value in 10 functions, no matter the mean value or standard deviation, and it performs better than other algorithms. And in the other eight functions, the optimal value of the mean can be found. For the index of 1/HV, MOWSO performs poorly. As can be seen from Table 5, MOWSO only finds the optimal value of mean or standard deviation in seven functions, and performs worse than several of them. In summary, the MOWSO algorithm performs better than the other five algorithms.

**Figure 3:**
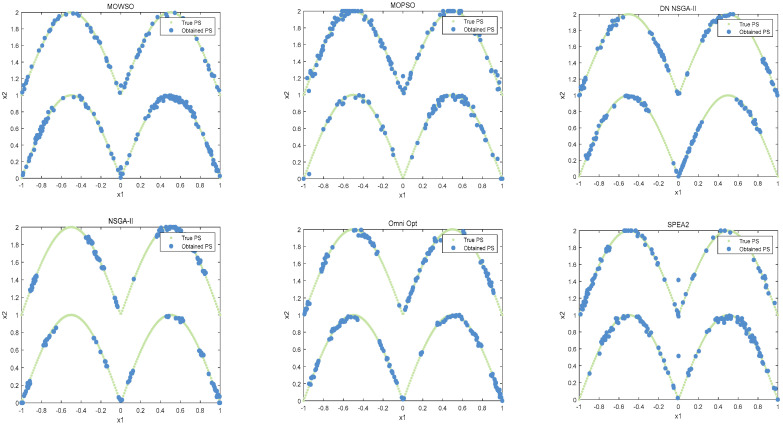
The Pareto set obtained by all algorithms on F3 (MMF4).

**Figure 4:**
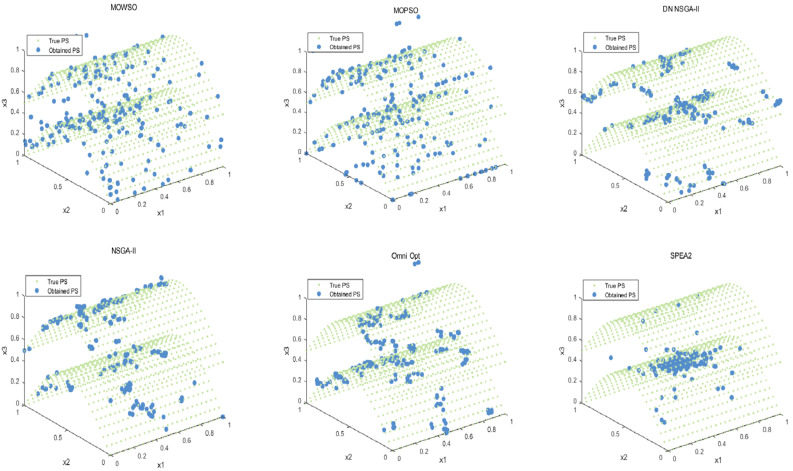
The Pareto set obtained by all algorithms on F14 (MMF14_a).

**Figure 5:**
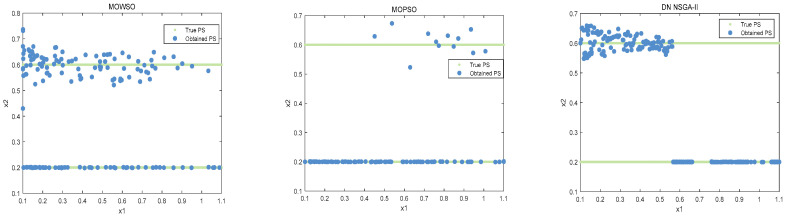

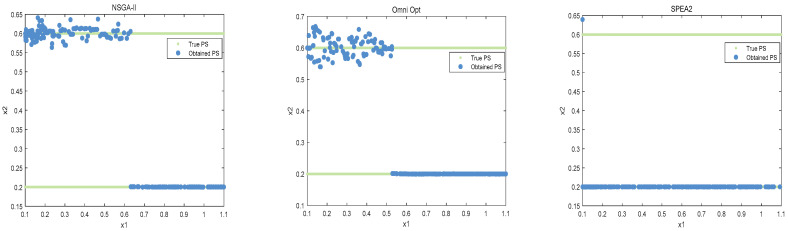
The Pareto set obtained by all algorithms on F16 (MMF10_l).

**Figure 6:**
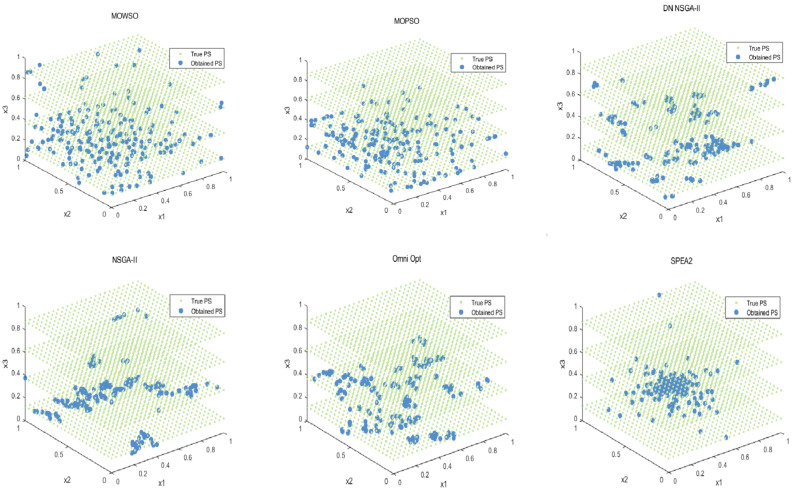
The Pareto set obtained by all algorithms on F24 (MMF16_l3).

**Figure 7:**
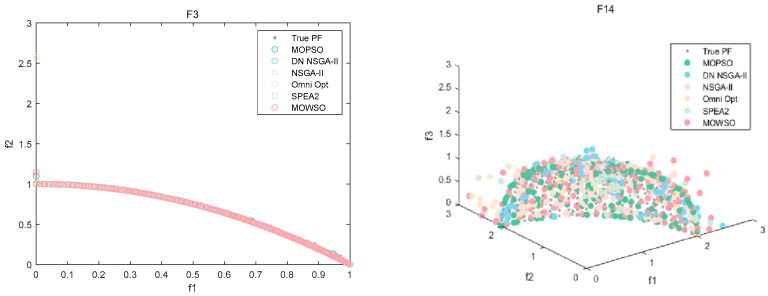

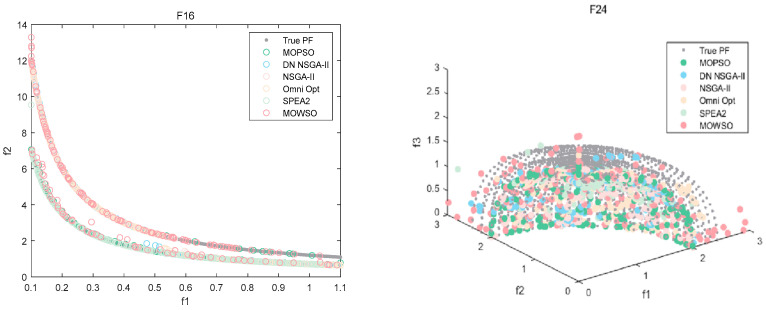
Pareto front of all algorithms on four functions.

**Figure 8:**
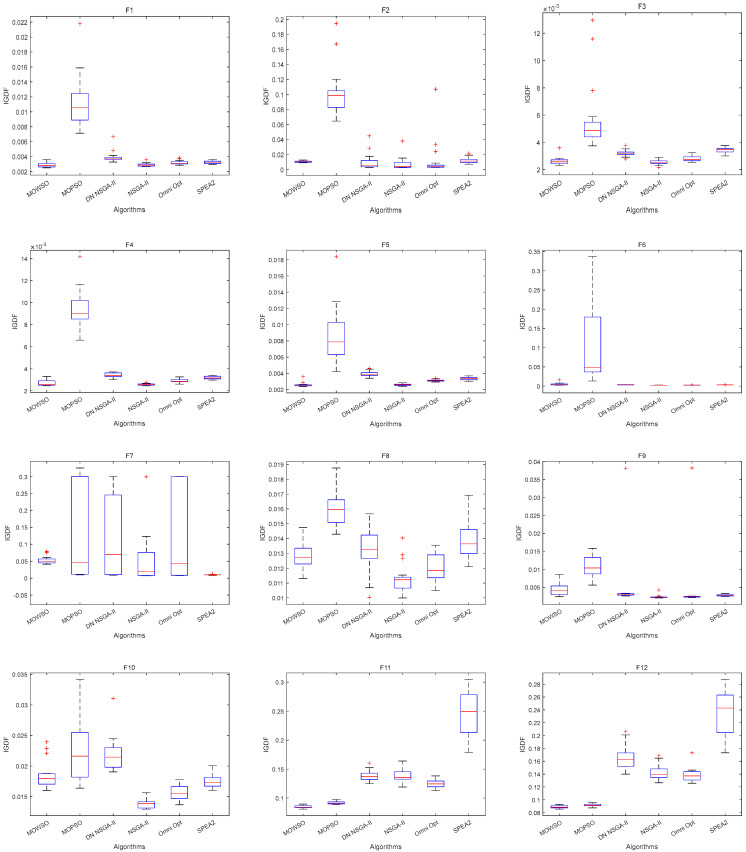

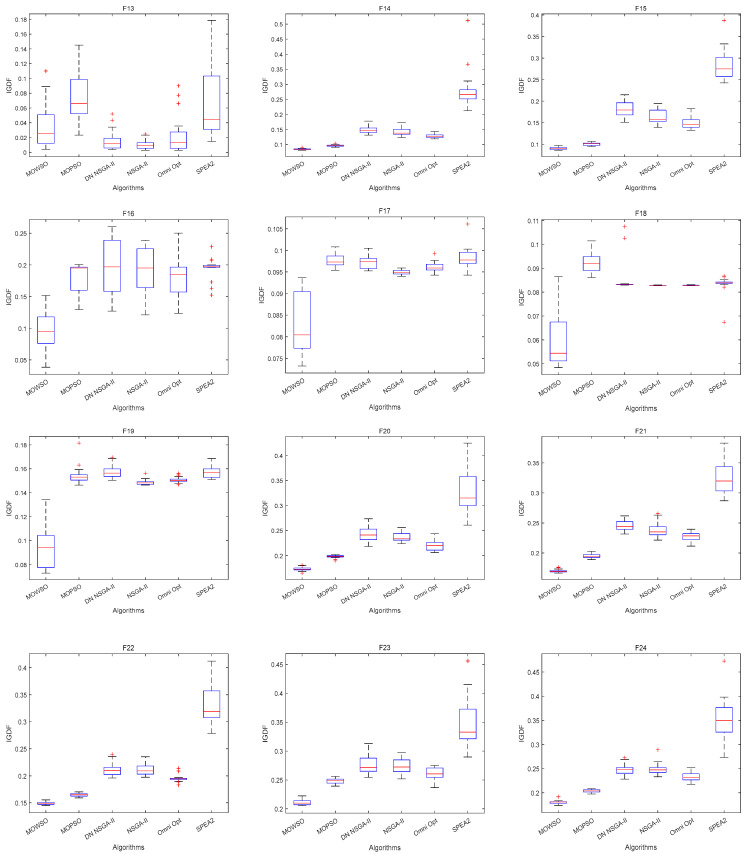
Comparison results (IGDF) of the box plot on CEC 2020 test functions.

The best results of PSs and PF were obtained by running each algorithm 20 times in functions F3, F14, F16 and F24. This is shown in Figure 3, Figure 4, Figure 5, Figure 6 and Figure 7. The number of dimensions and objective functions of the functions F3 and F16 is two, and the number of dimensions and objective functions of F14 and F24 is three [54]. As can be seen from Figure 3, the solution-set distribution obtained by the MOWSO algorithm on function F3 is more uniform; among the six functions, the solution set distribution of NSGA-II is more disorderly and the number of solutions obtained is less. As can be seen from Figure 4, on function F14, the MOWSO algorithm performs well, while SPEA2 obtains a poor distribution of solution sets. Figure 5 shows that on function F16, the solution set obtained by MOWSO is significantly better than the other five algorithms, and the solution-set distribution is more uniform than that obtained by the other five algorithms. As you can see from Figure 6, MOWSO also performs well on function F24. In Figure 7, it can be seen that the Pareto front obtained by MOWSO algorithm is widely distributed and uniform, indicating that the MOWSO algorithm is not easily trapped in local optimization when searching for solutions in the global. This paper presents drawings of the boxplot of IGDF on each function of different algorithms. The boxplot can display the distribution of data obtained by different algorithms intuitively. The box plot of the algorithm on the IGDF index is shown in Figure 8. It can be seen from the figure that MOWSO has the smallest upper and lower edges, upper and lower quartiles, and median on most functions compared with the other five algorithms. These results show that MOWSO performs stably and with minimal fluctuations on CEC 2020, and can reach optimal values compared to other algorithms.

Friedman rank can be used to compare the overall performance of different algorithms on data sets. In this paper, the performance of six algorithms on CEC 2020 was evaluated through a Friedman rank test experiment. The Friedman test obtains the average score and average ranking of each algorithm, thereby ranking the performance of the algorithm. Table 6 shows the results of the Friedman rank test for each algorithm. MOWSO’s overall ranking is No. 1. This proves the performance benefits of MOWSO.

## 5. Rural Sports-Facility Location Problem

### 5.1. The Objective Function of Sports-Facilities Location

In the location of traditional sports facilities, designers analyze various constraints, and constantly optimize and adjust the location scheme. Using the algorithm to solve this problem not only improves work efficiency and saves human resources, but also provides a new idea and method for the location and layout of sports facilities. When solving the location problem of sports facilities in real life, since sports facilities and villages occupy a relatively small area in the whole space, sports facilities (hereinafter referred to as facility points) and villages (hereinafter referred to as customers) are regarded as a point in the plane. According to the concept of a 15 min living circle, customers are required to walk 15 min to reach the location of the facility point. With the normal speed of 1.5 m/s as the standard, the effective service radius of the facility point is set to 1350 m. For the customer, the facility point is somewhere between expectation and exclusion. Customers not only want to be able to reach the facility point in a short time, but also because the facility point will bring certain congestion or other unfavorable factors, so customers do not want the facility point to be too close to them. Considering the maximum utilization of the facility point, it is hoped that there will be large number of customers within the effective coverage area of the facility point. This section introduces three objective functions in the continuous location of sports facilities. These objective functions are based on the classical model of a continuous facility location, and on this basis, is combined with practical problems to improve the objective functions.

The purpose of the classic multi-source Weber problem (MSWP) is to find the location of new facilities and minimize the sum of transportation costs between these new facilities and all customers. According to the customer’s hope to reach the service point in a short time, MSWP is selected as the first objective function. The mathematical model of this problem is as follows:

(29) $$\begin{array}{cc} MSWP: & \min\sum_{i=1}^{m}\sum_{j=1}^{n}w_{ij}\left\|x_{i}-a_{j}\right\|\\ s.t & \sum_{i=1}^{m}w_{ij}=s_{j},j=1,2,\ldots ,n,\\ & x_{i}\in R^{2},i=1,2,\ldots ,m, \end{array}$$

where m is the number of new facilities that need to be located. $x_{i}$ represents the position coordinate of the i-th facility point in the sought facility point. n represents the number of customers in the space. $a_{j}$ represents the location coordinates of the j-th customer. $w_{ij}$ indicates the weight between $x_{i}$ and $a_{j}$. ‖ ‖ is to calculate the Euclidean distance between two points.

In this model, each facility can provide enough services to customers in the target space, so that each customer can choose the nearest facility point to obtain services, thus minimizing the cost of the system. To avoid resource waste caused by too close a distance between sites’ selected points, constraints are added to the model, that is, the distance between two facility points should be greater than the minimum distance D. The model was adopted as the first objective function *F*_1_ of the sports-facility location problem. Finally, MSWP is equivalent to

(30) $$\begin{array}{l} F_{1}=\min\sum_{j=1}^{n}s_{j}\underset{i=1,\ldots m}{\min}\left\|x_{i}-a_{j}\right\|\\ s.t.\;\;\;\;\;x_{i}\in R^{2},i=1,2,\ldots ,m,\\ \;\;\;\;\;\left\|x_{p}-x_{q}\right\|>D,p,q=1,2,\ldots m, \end{array}$$

In order to avoid the waste of resources, more customers should exist within the effective coverage area of the facility, so that the facility can serve more customers. When there is a facility point within 1350 m from the customer, that is, the customer can reach the facility point within 15 min, it means that the customer is covered by the facility point. To enable more customers to be covered, the second objective function *F*_2_ uses a mathematical model as follows:

(31) $$\begin{array}{l} F_{2}=\max\sum_{j=1}^{n}z_{j}\\ s.t.\;\;\;\;\;z_{j}=\left\{\begin{array}{l} 1,\left\|a_{j}-x\right\|\leq R\\ 0,\left\|a_{j}-x\right\|>R \end{array}\right. \end{array}$$

Here, z=1 indicates that the customer has a facility point within a range of R, which ensures that the customer can walk to the facility point within 15 min. n is the number of customers.

Because the facilities will bring certain congestion or other adverse factors, customers want to be relatively far away from the facilities. Based on this, the distance of all customers to their nearest facility point is calculated. The customer closest to the facility point among all customers is expected to have the greatest distance from the facility point. Therefore, the third objective function *F*_3_ uses the Maximin model, as shown below:

(32) $$\begin{array}{l} F_{3}=\underset{x_{i}\in R^{2}}{\max}\;\;\underset{j=1,\ldots ,n}{\min}\;\;\underset{i=1,\ldots ,m}{\min}\left\|x_{i}-a_{j}\right\|\\ s.t.\;\;\;\;\;x_{i}\in R^{2},i=1,2,\ldots ,m,\\ \;\;\;\;\;\;\;\;\;\;\left\|x_{p}-x_{q}\right\|>D,p,q=1,2,\ldots m, \end{array}$$

where m represents the number of facility and $x_{i}$ represents the location of the i-th facility point. n is the number of customers and $a_{j}$ is the location of the j-th customer. ‖ ‖ is the Euclidean distance. D represents the minimum distance between two facility points.

### 5.2. Simulation Experiment Results

**Figure 9:**
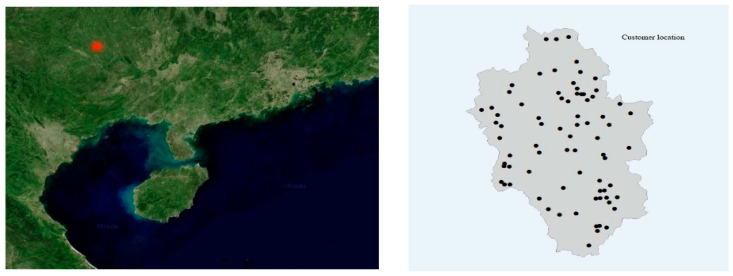
Case location and village distribution map.

This experiment uses a real case study, which is located in a township in Guangxi Province, China. ArcGIS was used to extract the village point coordinates of the township and it regarded them as customers, as shown in Figure 9. The number of customers involved in this case is 76, and the area of customers is about 120 square kilometers. A single sports facility has a service radius of 1350 m and covers an area of about 5.7 square kilometers. Considering the scattered distribution of customers, the number of experimental facilities in this experiment is nine facilities, and the number of facilities can be changed according to the actual situation. The problem is a multi-objective problem where F1 is the sum of the distances between all customers and the facility (SDCF), as shown in Equation (30). F2 is the number of customers covered by the sports facilities (NCCF), as shown in Equation (31). F3 is the distance between the nearest customer and the sports facility (DCCF), as shown in Equation (32).

**Figure 10:**
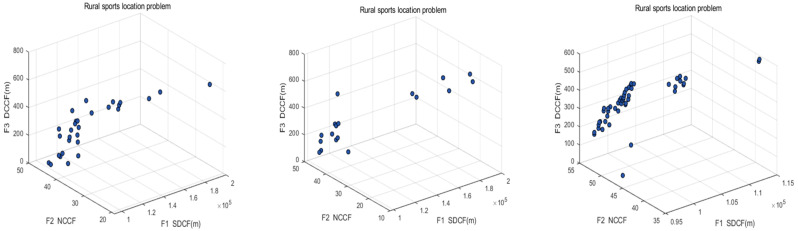
Location of sports facilities on the Pareto front.

The initial population of the MOWSO algorithm was set to 50 and the number of iterations to 100. Other MOWSO parameters are shown in Table 7. According to the three objective function models, MOWSO was used to solve the problem, and MOPSO and SPEA2 were compared. Finally, the location information of the facility point was obtained. The specific information of the results obtained by the three algorithms in each objective function is shown in Table 8. The Pareto frontier obtained by the solution is shown in Figure 10. From the Pareto front of the algorithm, it can be seen that the function values obtained by MOWSO are evenly distributed, which indicates that the MOWSO algorithm can provide more comprehensive and extensive reference information for the location problem of sports facilities and also proves the effectiveness of MOWSO algorithm.

**Figure 11:**
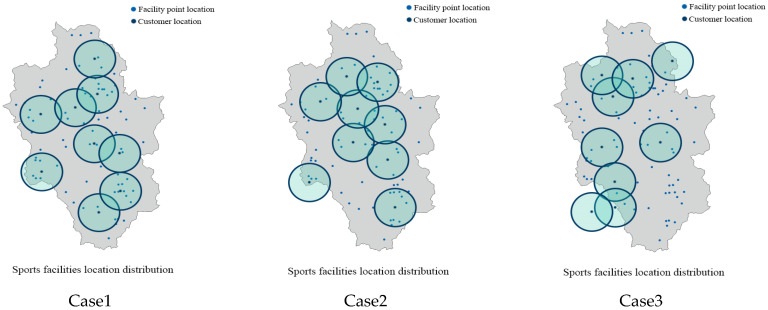
MOWSO obtained the distribution of sports facilities.

The specific information when MOWSO solves the location problem of sports facilities and obtains the best value in each objective function is shown in Table 9, and the specific location is shown in Figure 11. In the first case in the figure, the sum of the distances that all customers need to walk to reach the facility point is the minimum. At this time, the number of customers within the effective coverage of the facility is 42, and the distance between the customers closest to the sports facility and the facility is 84 m. In the second case, the number of customers within the effective coverage of the facility is the largest. At this time, all customers need to walk 112,556 m to reach the facility point, and the distance between the customers who are closest to the sports facility and the facility is 43 m. The third case is that the distance between the customers closest to the sports facilities and the facilities reaches the maximum. At this time, all customers need to walk 184,416 m to reach the facilities, and the number of customers within the effective coverage of the facilities is 19.

In order to verify the effectiveness of MOWSO in the location of sports facilities on a larger scale, 800 customer points were randomly generated in an area of 1225 square kilometers. The service radius of a single sports facility is 1350 m, covering an area of about 5.7 square kilometers, and the number of sports facilities is 100 to conduct experiments. According to the three objective function models, MOWSO was used to solve the problem, and MOPSO and SPEA2 were compared. The three algorithms were run separately ten times. Table 10 shows the average value of the optimal value obtained by the algorithm after ten times running on the three objective functions. F1 represents the sum of the distance that all customers need to travel to reach the facility point, in kilometers. F2 represents the number of customers within the effective coverage area of the facility. F3 is the distance between the nearest customer and the facility, in meters.

**Figure 12:**
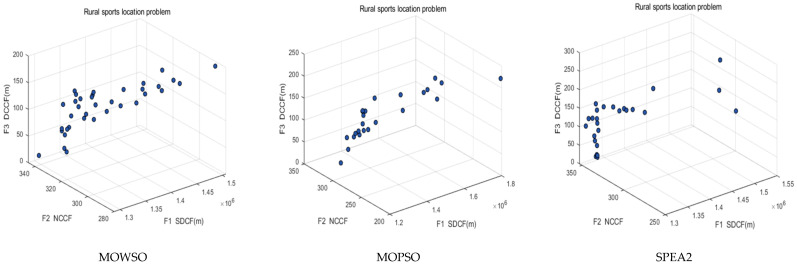
Pareto front of MOWSO, MOPSO, and SPEA2 algorithms.

The distribution of the solution was evaluated using the indices SP and UD, which are the diversity indices proposed by Schott and Tan et al. to measure the distribution of solutions [53,55]. The smaller the values of SP and UD, the better the distribution of the algorithm. The minimum value and average value of each algorithm running ten times were calculated, respectively, and the information is shown in Table 11. It can be seen from the table that MOWSO was run ten times on SP and UD indexes to obtain the smallest average value, indicating that the solution set obtained by the MOWSO algorithm has the best distribution. Figure 12 shows the Pareto front when each algorithm ran ten times to obtain the minimum value on UD. It can also be seen from the figure that the Pareto front of MOWSO is more evenly distributed.

## 6. Conclusions and Future Work

In this paper, we improve the white shark optimizer algorithm by adding two mechanisms. The first mechanism is to introduce the non-dominated Pareto solution obtained by archiving. The second mechanism is to calculate the true distance between Pareto solutions to filter the best Pareto solutions in the archive. The algorithm is tested on CEC2020 benchmark test function and compared with other classical multi-objective algorithms to verify the performance of MOWSO. The experimental results show that the Pareto solution obtained by MOWSO algorithm is closer to the real Pareto solution and has certain reliability. Then, MOWSO was applied to solve the location problem of rural sports facilities. Through the experiment, it was found that MOWSO can obtain multiple sets of solutions, corresponding to different layouts of sports facilities, and can provide reference for the location problem of rural sports facilities. In real life, there are many similar problems, such as the location of health stations, market sites, and so on. Although these problems have different constraints, similar ideas can be used to solve them. In this paper, the location of sports facilities is taken as a variable, and the location of sports facilities under different target conditions is studied carefully, but the terrain limitation is not considered. If the obtained sports facilities are located on non-construction land, such as paddy fields and agricultural land, etc., it is necessary to avoid these addresses manually. In real life, faced with these complex conditions, the constraints on the location of sports facilities will increase. Due to the high complexity of the specific problem, this experiment may not be considered enough. Therefore, it is very important to propose a new model and improve MOWSO to solve problems in this field under the condition of considering many factors comprehensively.

## Figures and Tables

**Table 1:** Literature review of metaheuristic algorithms.

Classification	Algorithm	Advantages	Improved Directing
Single objective/Multi-object	DE [4,5,6,7]	Simple principle, few controllable parameters, strong robustness	How to improve the optimization ability and convergence speed of the algorithm, and overcome the common defects of precocious convergence and search stagnation of heuristic algorithms
	PSO [8,9,10,11]	The convergence speed is fast, the parameters are few, and the algorithm is simple and easy to implement	There is a problem of becoming stuck in local optimal solution, so it relies on good initialization
	FA [12]	Effective for multimodal optimization and performs well when control parameters are adaptive	Highly sensitive to parameter settings and requires extensive tuning for complex problems
	ACO [13,14]	Robust for combinatorial problems, adaptable via pheromone information, and inspired by real ant behavior	Slow convergence and high computational complexity in large search spaces
	SCA [15,16]	Uses sine and cosine functions to explore and exploit search spaces effectively	May struggle with complex, highly constrained problems
	GWO [17,18,19]	Inspired by grey-wolf hierarchy and hunting mechanisms. Performs well on engineering design problems and benchmark function	Struggles with high-dimensional spaces compared to newer algorithm
	THRO [20]	The need for such a proposal arises from the limitations of existing optimization algorithms, which often struggle with convergence speed and solution accuracy when solving complex problems	THRO addresses these challenges by employing a unique dynamic individual matching strategy that enhances the algorithm’s convergence rate and solution precision
	DRA [21]	The DRA significantly outperforms other methods, delivering superior outcomes across multiple aspects, proving its efficacy in solving complex optimization problems	To explore the application of DRA in addressing optimization challenges across diverse scientific domains and practical scenarios, considering its potential to contribute to numerous future endeavors
	SOO [22]	SOO consistently outperforms its competitors across a wide variety of optimization tasks	SOO will focus on extending its capabilities to address a broader range of optimization challenges. The versatility of SOO opens doors to numerous application areas, particularly in machine learning and data analysis

**Table 2:** The mean and standard deviation of 1/PSP obtained by running the algorithm 20 times.

Number		MOWSO	MOPSO	DN NSGA-II	NSGA-II	Omni-Opt	SPEA2
F1	Mean	0.0524	0.1216	0.0859	0.1133	0.0830	0.0722
	Std	0.0059	0.0224	0.0141	0.0163	0.0141	0.0275
F2	Mean	0.0205	0.2218	0.0932	0.0804	0.1471	0.0726
	Std	0.0050	0.1546	0.0450	0.0228	0.1733	0.0588
F3	Mean	0.0364	0.0611	0.0961	0.1617	0.0846	0.0770
	Std	0.0106	0.0178	0.0151	0.0356	0.0211	0.0332
F4	Mean	0.1012	0.1879	0.1635	0.2060	0.1607	0.1253
	Std	0.0205	0.0269	0.0143	0.0313	0.0145	0.0372
F5	Mean	0.0368	0.0693	0.0478	0.0765	0.0408	0.0474
	Std	0.0120	0.0215	0.0080	0.0100	0.0092	0.0156
F6	Mean	0.3736	0.9456	0.2994	2.6998	0.3471	8.9296
	Std	0.7396	0.6040	0.1377	2.3663	0.1659	6.1643
F7	Mean	0.0144	0.1282	0.1152	0.0460	0.1356	0.0024
	Std	0.0034	0.1551	0.1336	0.0858	0.1640	0.0002
F8	Mean	0.0056	0.0049	0.0046	0.0033	0.0045	0.0033
	Std	0.0004	0.0003	0.0002	0.0004	0.0003	0.0004
F9	Mean	0.0029	0.0054	0.0040	0.0016	0.0038	0.0014
	Std	0.0007	0.0013	0.0078	0.0004	0.0079	0.0005
F10	Mean	0.0326	0.0737	0.0668	0.1139	0.0659	0.4046
	Std	0.0029	0.0560	0.0092	0.0421	0.0088	0.6052
F11	Mean	0.0673	0.0910	0.1282	0.1283	0.1123	0.2698
	Std	0.0021	0.0280	0.0108	0.0111	0.0081	0.0354
F12	Mean	0.0479	0.0479	0.0809	0.0749	0.0710	0.1076
	Std	0.0017	0.0020	0.0105	0.0064	0.0063	0.0138
F13	Mean	2.3580	5.4957	1.8312	3.1692	3.6821	11.6075
	Std	1.5159	4.9764	1.2674	1.7690	5.6489	7.3521
F14	Mean	0.0794	0.1037	0.1517	0.1632	0.1384	0.3530
	Std	0.0036	0.0114	0.0144	0.0150	0.0109	0.1802
F15	Mean	0.0544	0.0631	0.1090	0.1115	0.0939	0.1862
	Std	0.0013	0.0039	0.0144	0.0153	0.0150	0.0413
F16	Mean	0.0617	3.0757	3.2047	5.1204	3.772	3.2035
	Std	0.0356	2.5933	3.9127	6.6801	4.3618	1.5423
F17	Mean	0.5472	1.6414	1.6469	2.645	1.8104	3.3101
	Std	0.5158	0.2574	0.0799	0.6542	0.1086	2.0882
F18	Mean	0.5872	1.3375	2.0786	3.8000	2.2736	3.5535
	Std	0.6663	0.2587	0.5344	1.4332	0.1494	1.4231
F19	Mean	0.3440	0.5854	0.6099	0.7948	0.6164	1.3279
	Std	0.0866	0.0678	0.0206	0.1359	0.0474	0.7369
F20	Mean	0.1516	0.5913	0.3205	0.4557	0.3189	0.455
	Std	0.0157	0.0304	0.1426	0.2108	0.1402	0.1533
F21	Mean	0.1666	0.2738	0.2069	0.2829	0.2350	0.2991
	Std	0.0172	0.0198	0.0207	0.0296	0.0322	0.0485
F22	Mean	0.1226	0.2280	0.2304	0.2495	0.2277	0.2795
	Std	0.0064	0.0193	0.0447	0.0445	0.0380	0.0513
F23	Mean	0.1884	0.7838	0.3777	0.4095	0.4571	0.5305
	Std	0.0196	0.0739	0.1253	0.1956	0.1740	0.1430
F24	Mean	0.1548	0.3136	0.2753	0.3187	0.2970	0.3553
	Std	0.0093	0.0494	0.0296	0.0560	0.0423	0.0794

**Table 3:** The mean and standard deviation of IGDX obtained by running the algorithm 20 times.

Number		MOWSO	MOPSO	DN NSGA-II	NSGA-II	Omni-Opt	SPEA2
F1	Mean	0.0519	0.1192	0.0848	0.1108	0.0812	0.0704
	Std	0.0055	0.0215	0.0136	0.0155	0.0132	0.0246
F2	Mean	0.0198	0.1895	0.0865	0.0754	0.1162	0.0683
	Std	0.0044	0.0921	0.0402	0.0229	0.0980	0.0524
F3	Mean	0.0359	0.0608	0.0958	0.1560	0.0842	0.0742
	Std	0.0099	0.0174	0.0150	0.0332	0.0211	0.0295
F4	Mean	0.1001	0.1829	0.1604	0.2014	0.1584	0.1218
	Std	0.0198	0.0248	0.0137	0.0267	0.0139	0.0319
F5	Mean	0.0361	0.0631	0.0474	0.0739	0.0400	0.0448
	Std	0.0114	0.0164	0.0076	0.0087	0.0085	0.0134
F6	Mean	0.2561	0.7731	0.2894	1.3362	0.3382	2.3822
	Std	0.3594	0.3972	0.1301	0.5809	0.1616	0.6387
F7	Mean	0.0140	0.1261	0.1125	0.0441	0.1339	0.0024
	Std	0.0032	0.1526	0.1327	0.0851	0.1642	0.0002
F8	Mean	0.0056	0.0049	0.0046	0.0033	0.0045	0.0033
	Std	0.0004	0.0003	0.0002	0.0004	0.0003	0.0004
F9	Mean	0.0029	0.0054	0.0040	0.0016	0.0038	0.0014
	Std	0.0007	0.0013	0.0078	0.0004	0.0079	0.0005
F10	Mean	0.0323	0.0936	0.0667	0.0957	0.0657	0.1342
	Std	0.0027	0.0593	0.0091	0.0182	0.0087	0.0425
F11	Mean	0.0673	0.0910	0.1282	0.1283	0.1123	0.2609
	Std	0.0021	0.0280	0.0108	0.0111	0.0081	0.0315
F12	Mean	0.0479	0.0476	0.0809	0.0749	0.0710	0.1033
	Std	0.0017	0.0020	0.0105	0.0064	0.0063	0.0107
F13	Mean	1.3629	2.1221	1.1746	1.7155	1.4404	2.9145
	Std	0.6273	0.8221	0.5368	0.6044	0.8486	0.5991
F14	Mean	0.0793	0.1037	0.1517	0.1630	0.1383	0.2651
	Std	0.0036	0.0114	0.0144	0.0151	0.0109	0.0708
F15	Mean	0.0543	0.0631	0.1090	0.1115	0.0939	0.1627
	Std	0.0012	0.0039	0.0144	0.0153	0.0150	0.0166
F16	Mean	0.0611	0.1772	0.1819	0.1777	0.1770	0.1967
	Std	0.0352	0.0446	0.0377	0.0388	0.0403	0.0114
F17	Mean	0.2146	0.2492	0.2504	0.2513	0.2503	0.2509
	Std	0.0266	0.0008	0.0002	0.0004	0.0004	0.0019
F18	Mean	0.1826	0.2446	0.2440	0.2472	0.2464	0.2455
	Std	0.0460	0.0015	0.0116	0.0004	0.0002	0.0045
F19	Mean	0.2398	0.2806	0.2910	0.3125	0.2885	0.3511
	Std	0.0177	0.0196	0.0097	0.0116	0.0115	0.0273
F20	Mean	0.1516	0.2668	0.2458	0.2677	0.2440	0.2955
	Std	0.0157	0.0018	0.0266	0.0252	0.0252	0.0222
F21	Mean	0.1640	0.2266	0.2043	0.2441	0.2191	0.2519
	Std	0.0136	0.0063	0.0159	0.0080	0.0142	0.0211
F22	Mean	0.1226	0.1756	0.2038	0.2143	0.1990	0.2388
	Std	0.0064	0.0118	0.0141	0.0130	0.0094	0.0160
F23	Mean	0.1883	0.3381	0.3023	0.3081	0.3074	0.3453
	Std	0.0193	0.0026	0.0252	0.0364	0.0342	0.0226
F24	Mean	0.1548	0.2309	0.2442	0.2619	0.2463	0.2779
	Std	0.0093	0.0280	0.0115	0.0165	0.0124	0.0284

**Table 4:** The mean and standard deviation of IGDF obtained by running the algorithm 20 times.

Number		MOWSO	MOPSO	DN NSGA-II	NSGA-II	Omni-Opt	SPEA2
F1	Mean	0.0029	0.0113	0.0039	0.0029	0.0032	0.0033
	Std	0.0003	0.0034	0.0007	0.0002	0.0003	0.0002
F2	Mean	0.0104	0.1008	0.0104	0.0073	0.0116	0.0121
	Std	0.0010	0.0315	0.0105	0.0082	0.0238	0.0038
F3	Mean	0.0026	0.0057	0.0032	0.0026	0.0028	0.0034
	Std	0.0003	0.0024	0.0002	0.0002	0.0002	0.0002
F4	Mean	0.0027	0.0093	0.0034	0.0027	0.0029	0.0032
	Std	0.0003	0.0017	0.0002	0.0001	0.0002	0.0001
F5	Mean	0.0026	0.0088	0.0039	0.0026	0.0031	0.0033
	Std	0.0003	0.0034	0.0003	0.0001	0.0001	0.0002
F6	Mean	0.0050	0.1093	0.0037	0.0026	0.0030	0.0033
	Std	0.0032	0.1054	0.0003	0.0001	0.0001	0.0002
F7	Mean	0.0529	0.1325	0.1253	0.0526	0.1274	0.0101
	Std	0.0116	0.1351	0.118	0.0692	0.1333	0.0009
F8	Mean	0.0128	0.0160	0.0132	0.0113	0.0120	0.0139
	Std	0.0009	0.0013	0.0015	0.0010	0.0010	0.0012
F9	Mean	0.0043	0.0108	0.0047	0.0023	0.0042	0.0028
	Std	0.0016	0.0028	0.0079	0.0005	0.0080	0.0003
F10	Mean	0.0184	0.0226	0.0219	0.0138	0.0157	0.0176
	Std	0.0021	0.0049	0.0027	0.0007	0.0012	0.0012
F11	Mean	0.0847	0.0918	0.1383	0.1378	0.1245	0.2457
	Std	0.0023	0.0025	0.0088	0.0105	0.0068	0.0388
F12	Mean	0.0886	0.0914	0.1656	0.1426	0.1381	0.2355
	Std	0.0021	0.0022	0.0189	0.0115	0.0107	0.0344
F13	Mean	0.0350	0.0730	0.0159	0.0103	0.0229	0.0665
	Std	0.0297	0.0305	0.0135	0.0067	0.0256	0.0478
F14	Mean	0.0843	0.0965	0.1489	0.1415	0.1281	0.2797
	Std	0.0024	0.0029	0.0125	0.0118	0.0060	0.0642
F15	Mean	0.0902	0.1003	0.1821	0.1640	0.1492	0.2819
	Std	0.0027	0.0033	0.0191	0.0160	0.0137	0.0353
F16	Mean	0.0967	0.1764	0.1965	0.1917	0.1813	0.1951
	Std	0.0278	0.0244	0.0420	0.0400	0.0363	0.0161
F17	Mean	0.0829	0.0977	0.0972	0.0949	0.0961	0.0983
	Std	0.0072	0.0014	0.0014	0.0005	0.0011	0.0025
F18	Mean	0.0606	0.0924	0.0854	0.0828	0.0829	0.0833
	Std	0.0121	0.0042	0.0068	0.0001	0.0001	0.0039
F19	Mean	0.0948	0.1543	0.1579	0.1487	0.1507	0.1570
	Std	0.0185	0.0076	0.0058	0.0022	0.0023	0.0047
F20	Mean	0.1735	0.1982	0.2439	0.2373	0.2198	0.3295
	Std	0.0038	0.0028	0.0144	0.0094	0.0095	0.0449
F21	Mean	0.1707	0.1951	0.2460	0.2384	0.2272	0.3261
	Std	0.0029	0.0032	0.0085	0.0120	0.0079	0.0284
F22	Mean	0.1489	0.1648	0.2115	0.2114	0.1951	0.3297
	Std	0.0026	0.0032	0.0124	0.0107	0.0064	0.0363
F23	Mean	0.2110	0.2480	0.2755	0.2736	0.2610	0.3465
	Std	0.0051	0.0051	0.0140	0.0128	0.0104	0.0407
F24	Mean	0.1801	0.2037	0.2478	0.2489	0.2328	0.3497
	Std	0.0038	0.0032	0.0109	0.0125	0.0088	0.0459

**Table 5:** The mean and standard deviation of 1/HV obtained by running the algorithm 20 times.

Number		MOWSO	MOPSO	DN NSGA-II	NSGA-II	Omni-Opt	SPEA2
F1	Mean	1.1465	1.3347	1.1485	1.1466	1.1472	1.1475
	Std	0.0008	0.3178	0.0013	0.0009	0.0008	0.0006
F2	Mean	1.1651	1.3838	1.1583	1.1535	1.1575	1.1829
	Std	0.0026	0.0798	0.0140	0.0102	0.0264	0.0536
F3	Mean	1.8561	1.0105	1.8581	1.8530	1.8552	1.8593
	Std	0.0030	3.0166	0.0009	0.0006	0.0009	0.0023
F4	Mean	1.1462	1.1742	1.1481	1.1457	1.1466	1.1475
	Std	0.0007	0.0578	0.0012	0.0004	0.0005	0.0005
F5	Mean	1.1458	1.4250	1.1495	1.1457	1.1470	1.1473
	Std	0.0007	0.2880	0.0011	0.0003	0.0003	0.0005
F6	Mean	2.4015	13.3576	2.3822	2.3715	2.3743	2.4049
	Std	0.0286	55.8386	0.0036	0.0006	0.0006	0.0175
F7	Mean	0.0810	0.0812	0.0807	0.0795	0.0808	0.0777
	Std	0.0011	0.0039	0.0032	0.0026	0.0038	0.0000
F8	Mean	0.0689	0.0689	0.0689	0.0689	0.0689	0.0689
	Std	0.0000	0.0000	0.0000	0.0000	0.0000	0.0000
F9	Mean	0.6372	0.6417	0.6447	0.6356	0.6445	0.6365
	Std	0.0015	0.0033	0.0400	0.0005	0.0401	0.0010
F10	Mean	0.0543	0.0543	0.0543	0.0543	0.0543	0.0543
	Std	0.0000	0.0000	0.0000	0.0000	0.0000	0.0000
F11	Mean	0.3856	0.3538	0.3275	0.3555	0.3392	0.4043
	Std	0.0352	0.0118	0.0172	0.0085	0.0148	0.0611
F12	Mean	0.2543	0.2399	0.2378	0.2396	0.2381	0.2697
	Std	0.0085	0.0067	0.0168	0.0074	0.0115	0.0254
F13	Mean	1.2210	0.8559	6.5633	1.1607	1.1723	1.1734
	Std	0.0828	2.8956	8.5302	0.0102	0.0269	0.4056
F14	Mean	0.3835	0.3361	0.3172	0.3486	0.3321	0.4115
	Std	0.0202	0.0140	0.0183	0.0091	0.0131	0.0851
F15	Mean	0.2544	0.2331	0.2352	0.2323	0.2362	0.2746
	Std	0.0154	0.0060	0.0102	0.0056	0.0118	0.0283
F16	Mean	0.0815	0.0809	0.0809	0.0800	0.0808	0.0779
	Std	0.0010	0.0040	0.0029	0.0025	0.0031	0.0007
F17	Mean	0.0689	0.0689	0.0689	0.0689	0.0689	0.0689
	Std	0.0000	0.0000	0.0000	0.0000	0.0000	0.0000
F18	Mean	0.6377	0.6426	0.6453	0.6355	0.6356	0.6365
	Std	0.0014	0.0045	0.0400	0.0001	0.0000	0.0012
F19	Mean	0.0543	0.0543	0.0543	0.0543	0.0543	0.0543
	Std	0.0000	0.0001	0.0000	0.0000	0.0000	0.0000
F20	Mean	0.2558	0.2367	0.2407	0.2386	0.2356	0.2674
	Std	0.0099	0.0066	0.0147	0.0068	0.0118	0.0314
F21	Mean	0.2562	0.2360	0.2430	0.2349	0.2327	0.2707
	Std	0.0115	0.0054	0.0195	0.0063	0.0085	0.0338
F22	Mean	0.2517	0.2323	0.2306	0.2339	0.2312	0.2692
	Std	0.0116	0.0053	0.0091	0.0056	0.0063	0.0325
F23	Mean	0.2538	0.2326	0.2314	0.2337	0.2350	0.2763
	Std	0.0108	0.0068	0.0102	0.0058	0.0088	0.038
F24	Mean	0.2544	0.2341	0.2329	0.2350	0.2312	0.2653
	Std	0.0166	0.0061	0.0128	0.0058	0.0090	0.0311

**Table 6:** Friedman rank test.

Algorithm	1/PSP		IGDX		IGDF		1/HV		Result	
	Score	Rank	Score	Rank	Score	Rank	Score	Rank	Score	Rank
MOWSO	1.5000	1	1.4167	1	1.9167	1	4.2083	5	2.2604	1
MOPSO	3.8750	4	3.6250	4	4.2083	4	3.8542	4	3.8906	5
NSGA-II	3.3750	3	3.5833	3	4.5833	5	3.3125	3	3.7135	4
DN_ NSGA-II	4.4583	5	4.6042	6	2.5000	2	2.2500	1	3.4531	3
Omni-Opt	3.3333	2	3.2500	2	3.0000	3	2.8333	2	3.1042	2
SPEA2	4.4583	6	4.5208	5	4.7017	6	4.5417	6	4.5781	6

**Table 7:** Setting of algorithm parameters.

Algorithm	Parameters					
	Population	τ	$f_{\max}$	$f_{\min}$	$p_{\max}$	$p_{\min}$
MOWSO	50	4.125	0.75	0.07	1.5	0.5

**Table 8:** Partial Pareto optimal solution.

Objective Function	Algorithm								
	MOWSO			MOPSO			SPEA2		
	F1_Min	F2_Max	F3_Max	F1_Min	F2_Max	F3_Max	F1_Min	F2_Max	F3_Max
F1	92,801	112,556	184,416	96,882	10,202	186,539	94,981	95,258	114,161
F2	42	46	19	43	45	18	45	52	38
F3	84	43	693	134	184	702	70	207	603

**Table 9:** Pareto optimal solution obtained by MOWSO.

Solution	Position Coordinate	Facility Point Number									Objective Function Value		
		1	2	3	4	5	6	7	8	9	F1	F2	F3
1	x	7393	1949	2309	5587	7639	4205	5206	6318	5629	93,805	46	74
	y	6038	8433	4355	10,118	3380	9058	12,603	1750	6574			
2	x	4659	2171	6945	5820	7686	6563	1841	3763	4539	102,614	48	195
	y	8979	9328	5572	10,949	2291	8014	3642	11,196	6629			
3	x	2464	4001	3289	6651	7027	4461	3835	2439	2793	155,106	25	633
	y	1643	2051	9725	6781	12,467	11,076	3850	11,148	6162			

**Table 10:** The algorithm obtains the value of the objective function.

Objective Function	Algorithm								
	MOWSO			MOPSO			SPEA2		
	F1	F2	F3	F1	F2	F3	F1	F2	F3
Mean	1276	351	205	1310	339	223	1278	361	219

**Table 11:** The average of 10 runs of the algorithm on SP and UD.

Evaluation	Algorithm					
	MOWSO		MOPSO		SPEA2	
	Min	Mean	Min	Mean	Min	Mean
SP	2015	5754	9524	219.67	2095	16,643
UD	0.1175	0.2113	0.1475	0.2277	0.1119	0.2328

## Data Availability

The original contributions presented in the study are included in the article. Further inquiries can be directed to the corresponding authors.
